# Supplementary material for: Substrate thermal properties influence ventral brightness evolution in ectotherms
Source: Commun Biol. 2021 Jan 4;4:26. doi: 10.1038/s42003-020-01524-w (PMC7782800; doi:10.1038/s42003-020-01524-w)
Supplement: Supplementary file 1 — Supplementary Information [file 42003_2020_1524_MOESM1_ESM.pdf]

Supplementary Material

All R-scripts, datasets and the macro developed for ImageJ are available at the provided repository[1].

Table S.1. In this study we classified the substrate type on which species are found following this table. We first retrieved information of where species are thriving from literature (all references are provided in the supplied repository). We then investigated the natural history and ecological background of the species. Following, based on the description of the habitat type, we identified six different categories (A-F). The categories reflect a Specific Heat Capacity (c_p) gradient from A = low c_p (i.e. hot grounds), to F = high c_p (i.e. cool grounds). C_p values retrieved from engineeringtoolbox.com (www.engineeringtoolbox.com/specific-heat-solids-d_154.html; www.engineeringtoolbox.com/specific-heat-capacity-d_391.html).

**Category**	**A**	**B**	**C**	**D**	**E**	**F**
**Description**	Arid substrates (e.g. sandy, rocky grounds)	Arid substrates in combination with forest grounds and/or grass patches	Species inhabiting more than three different substrate categories	Grass patches in combination with forest grounds	Arboreal (vegetation higher than 5 m)	Forest grounds
**Examples (reported c_p values in *kcal/(kg °C)*)**	Basalt rock (0.2), Dolomite rock (0.22), Granite (0.19), Sand dry (0.19), Sandstone (0.22), Limestone (0.217), Soil dry (0.19)	**Any** arid substrate falling into category A **+** **Any** of Soil wet (0.35), Grass (0.382-0.502), **and/or** Wet mud (0.60)	**Any combination of** 4 (or more) category types	Soil wet (0.35), Grass (0.382-0.502) **+** Wet mud (0.60)	Wood oak (0.48), Wood white pine (0.6), Wood balsa (0.7)	Wet mud (0.60), Water (1.00)

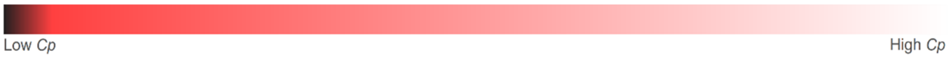

We categorized substrates based on literature information (provided in the supplied repository) on habitat type and ecology of species. Our classification reflects biologically relevant substrates on which the studied species can be found.

Despite category E (high vegetation) and F (forest grounds) display similar substrate specific heat capacity (c_p) values, the species ecology and the environmental pressures individuals experience on those substrates are likely different. Therefore, for this analysis we separated those two categories.

Category B includes any of the arid substrates falling within category A plus any substrate falling within category D. Category C includes at least four different substrate categories. We classified it between category B and D because no matter which category combination is considered, the overall heat stress from the ground is lower than the hotter substrates (i.e. A-B) and higher than the cooler ones (D-F). A reader can argue that category C is redundant and should be incorporated within e.g. category B or D. However, the selection pressures species have to endure in multiple substrates are likely different from those who thrive on limited amounts, hence the addition of category C. As an example, *Protobothrops mucrosquamatus* can be found on grasslands, forest grounds, shrublands, around human settlements and agricultural lands, and have arboreal tendencies, thus we classified it within category C (https://www.iucnredlist.org/species/178409/7540882; http://www.toxinology.com/fusebox.cfm?fuseaction=main.snakes.display&id=SN0111).

We followed Dehgan, B. [2] 5 m threshold height definition for a tree, whereas shrubs are generally having a height of 1-5 m and multiple trunks.

Table S.2. The 29 squamate species from the Tel Aviv University's Garden for Zoological Research used to assess the relationship between spectrophotometry data and image brightness obtained from our custom-made macro in ImageJ and the relationship between the visible and near-Infrared spectra. The step-by-step procedure to acquire brightness values through the interactive macro is provided in the main text with a graphical explanation in Figure S.1.

**Species**	**Family**
Cerastes cerastes	Viperidae
Cerastes gasperettii	Viperidae
Chalcides guentheri	Scincidae
Chalcides ocellatus	Scincidae
Chalcides sepsoides	Scincidae
Daboia palaestinae	Viperidae
Dolichophis jugularis	Colubridae
Echis coloratus	Viperidae
Eirenis decemlineatus	Colubridae
Elaphe sauromates	Colubridae
Eryx jaculus	Boidae
Eublepharis macularius	Eublepharidae
Eumeces schneiderii	Scincidae
Heloderma suspectum	Helodermatidae
Hemorrhois nummifer	Colubridae
Iguana iguana	Iguanidae
Malpolon insignitus	Lamprophiidae
Mediodactylus kotschyi	Gekkonidae
Natrix tessellata	Colubridae
Phoenicolacerta laevis	Lacertidae
Platyceps collaris	Colubridae
Platyceps rogersi	Colubridae
Pogona vitticeps	Agamidae
Ptyodactylus guttatus	Phyllodactylidae
Scincus scincus	Scincidae
Spalerosophis diadema	Colubridae
Stenodactylus doriae	Gekkonidae
Trachylepis vittata	Scincidae
Walterinnesia aegyptia	Elapidae

Table S.3. Picture mean brightness and standard deviation (SD) of the 29 squamate species used in the validation analysis between spectrophotometry and image brightness (Figure S.4). A graphical representation is provided in Figure S.5. Mean number of pictures per species = 9.81, SD = 1.41.

**Species**	**Mean (Image Brightness)**	**SD**
Cerastes cerastes	41.16	11.57
Cerastes gasperettii	44.36	11.02
Chalcides guentheri	37.44	13.06
Chalcides ocellatus	35.86	9.18
Chalcides sepsoides	40.86	14.77
Daboia palaestinae	28.57	8.70
Dolichophis jugularis	32.58	13.44
Echis coloratus	40.56	10.54
Eirenis decemlineatus	33.67	9.78
Elaphe sauromates	37.29	10.35
Eryx jaculus	34.73	9.98
Eublepharis macularius	32.94	9.82
Eumeces schneideri	39.98	15.26
Heloderma suspectum	33.67	7.15
Hemorrhois nummifer	33.94	9.34
Iguana iguana	40.70	9.58
Malpolon insignitus	26.27	8.60
Mediodactylus kotschyi	32.46	9.44
Natrix tessellata	28.03	9.23
Phoenicolacerta laevis	35.12	8.91
Platyceps collaris	32.64	10.02
Platyceps rogersi	37.02	10.13
Pogona vitticeps	33.90	10.02
Ptyodactylus guttatus	39.71	11.01
Scincus scincus	39.96	14.55
Spalerosophis diadema	39.20	13.27
Stenodactylus doriae	40.30	14.90
Trachylepis vittata	34.65	9.31
Walterinnesia aegyptia	15.44	6.41

Table S.4. Model selection output of MCMCglmm Ventral ~ -1 + Substrate + Dorsal + Altitude + Distribution + Log body mass + Polymorphic + Day cycle. Only mostly supported models (DIC <5) are reported for graphical interpretation. Model = Model number from the MCMCglmm chain, Alt=Altitude, Dst=Distribution, Drs=Dorsal, Log.bdy.mss=Log body mass, Ply=Polymorphic, Sbs=Substrate.

Model	Alt	Dst	Drs	Log.bdy.mss	Ply	Sbs	df	logLik	DIC	delta	weight
86	+	+		3.758		+	17	-444.22	905.3	0	0.358
85		+		3.556		+	13	-447.125	907.1	1.84	0.143
118	+	+		3.701	+	+	18	-444.687	907.2	1.93	0.136
94	+	+	-0.00673	3.761		+	18	-444.824	907.5	2.18	0.12
117		+		3.504	+	+	14	-446.925	907.7	2.42	0.107
93		+	-0.04737	3.546		+	14	-447.632	909.1	3.81	0.053
126	+	+	-0.00469	3.713	+	+	19	-445.273	909.4	4.1	0.046
125		+	-0.02378	3.498	+	+	15	-447.494	909.8	4.52	0.037

Table S.5. Cumulative Akaike weights of the parameters employed in the ventral full models. Sbs=Substrate, Dst=Distribution, Log.bdy.mss=Log body mass, Alt=Altitude, Ply=Polymorphic, Drs=Dorsal, Day.cyc=Day cycle.

	Sbs	Dst	Log.bdy.mss	Alt	Ply	Drs	Day.cyc
**Sum of weights:**	1	1	0.94	0.65	0.33	0.26	0.06
**N containing models:**	64	64	64	64	64	64	64

Table S.6. Output of the MCMCglmm model: Ventral ~ Substrate + Distribution + Log body mass. DIC = 907.12. Reference level: Substrate A + Distribution Subtropical. Substrate A = arid substrates, Substrate B = arid substrates + grass +/ forest grounds, Substrate C = generalists, Substrate D = grass + forest grounds, Substrate E = high vegetation, Substrate F = forest grounds.

	post.mean	l-95% CI	u-95% CI	eff.samp	pMCMC	
**(Intercept)**	64.64	56.29	73.11	48000	< 2e-05	***
**SubstrateB**	-16.25	-22.19	-10.43	48000	< 2e-05	***
**SubstrateC**	-21.13	-27.37	-15.18	48000	< 2e-05	***
**SubstrateD**	-20.65	-27.77	-13.87	48352	< 2e-05	***
**SubstrateE**	-24.08	-31.13	-17.02	48000	< 2e-05	***
**SubstrateF**	-20.63	-27.49	-13.59	49244	< 2e-05	***
**DistributionSubtropical-Temperate**	-10.35	-16.86	-4.06	48000	0.0019	**
**DistributionTemperate**	-9.12	-14.86	-3.19	48000	0.0025	**
**DistributionTemperate-Polar**	-14.58	-31.60	2.62	48854	0.0930	.
**DistributionTropical**	0.94	-3.74	5.76	48000	0.6961	
**DistributionTropical-Subtropical**	1.04	-3.82	6.01	48000	0.6773	
**Log.body.mass**	3.55	0.90	6.28	48000	0.0105	*

Signif. codes: 0 ‘***’ 0.001 ‘**’ 0.01 ‘*’ 0.05 ‘.’ 0.1 ‘ ’ 1

Table S.7. Reconstructed posterior means [lower, upper 95% credible intervals] of the ventral brightness from the output of the MCMCglmm model in Table S.5. Substrate A = arid substrates, Substrate B = arid substrates + grass +/ forest grounds, Substrate C = generalists, Substrate D = grass + forest grounds, Substrate E = high vegetation, Substrate F = forest grounds.

	Distribution						
**Body Mass = 0**	**Tropical**	**Tropical-Subtropical**	**Subtropical**	**Subtropical-Temperate**	**Temperate**	**Temperate-Polar**	**Substrate Mean**
**SubstrateA**	65.58 [52.55,78.87]	65.68 [52.47,79.12]	64.64 [56.29,73.11]	54.30 [39.43,69.05]	55.52 [41.42,69.92]	50.06 [24.68,75.73]	59.30 [44.47,74.30]
**SubstrateB**	49.33 [30.36,68.44]	49.43 [30.28,68.69]	48.40 [34.10,62.68]	38.05 [17.24,58.62]	39.27 [19.23,59.49]	33.81 [2.50,65.30]	43.05 [22.29,63.87]
**SubstrateC**	44.45 [25.17,63.68]	44.55 [25.09,63.94]	43.51 [28.91,57.93]	33.17 [12.05,53.86]	34.39 [14.05,54.74]	28.93 [-2.69,60.55]	38.17 [17.10,59.12]
**SubstrateD**	44.93 [24.78,65.00]	45.03 [24.70,65.26]	44.00 [28.52,59.25]	33.65 [11.66,55.18]	34.87 [16.66,56.06]	29.41 [-3.08,61.86]	38.65 [16.71,60.43]
**SubstrateE**	41.50 [21.42,61.84]	41.60 [21.34,62.10]	40.57 [25.16,56.09]	30.22 [8.30,52.02]	31.44 [10.29,52.90]	25.98 [-6.45,58.71]	35.22 [13.34,57.28]
**SubstrateF**	44.95 [25.06,65.28]	45.05 [24.98,65.54]	44.02 [28.80,59.52]	33.67 [11.94,55.46]	34.89 [13.93,56.34]	29.43 [-2.80,62.14]	38.67 [16.99,60.71]
**Distribution Mean**	48.46 [34.57,62.37]	48.56 [34.67,62.47]	47.52 [33.63,61.43]	37.17 [23.28,51.08]	38.40 [24.51,52.31]	32.94 [19.04,46.84]	
**Body Mass = 1**							
**SubstrateA**	69.13 [53.45,85.14]	69.23 [53.37,85.40]	68.19 [57.19,79.39]	57.85 [40.33,75.32]	59.07 [42.33,76.20]	53.61 [25.59,82.01]	62.85 [45.38,80.58]
**SubstrateB**	52.88 [31.27,74.71]	52.98 [31.19,74.97]	51.95 [35.00,68.96]	41.60 [18.15,64.89]	42.83 [20.14,65.77]	37.36 [3.40,71.58]	46.60 [23.19,70.15]
**SubstrateC**	48.00 [26.08,69.96]	48.10 [26.00,70.22]	47.06 [29.82,64.20]	36.72 [12.96,60.14]	37.94 [14.95,61.02]	32.48 [-1.79,66.82]	41.72 [18.00,65.39]
**SubstrateD**	48.48 [25.69,71.28]	48.58 [25.61,71.53]	47.55 [29.42,65.52]	37.20 [12.57,61.46]	38.43 [14.56,62.33]	32.96 [-2.18,68.14]	42.20 [17.61,66.71]
**SubstrateE**	45.06 [22.32,68.12]	45.16 [22.24,68.38]	44.12 [26.06,62.36]	33.77 [9.20,58.30]	35.00 [11.20,59.17]	29.53 [-5.54,64.98]	38.77 [14.25,63.55]
**SubstrateF**	48.50 [25.97,71.56]	48.60 [25.89,71.81]	47.57 [29.70,65.80]	37.22 [12.85,61.74]	38.45 [14.84,62.61]	32.98 [-1.90,68.42]	42.22 [17.89,66.99]
**Distribution Mean**	52.01 [30.80,73.46]	52.11 [30.72,73.72]	51.07 [34.53,67.71]	40.73 [17.68,63.64]	41.95 [19.67,64.52]	36.49 [2.93,70.32]	

Table S.8. Output of the graphical MCMCglmm model Ventral ~ -1 + Substrate | Distribution + Log body mass. DIC= 912.99. Substrate A = arid substrates, Substrate B = arid substrates + grass +/ forest grounds, Substrate C = generalists, Substrate D = grass + forest grounds, Substrate E = high vegetation, Substrate F = forest grounds.

	**post.mean**	**l-95% CI**	**u-95% CI**	**eff.samp**	**pMCMC**	
**SubstrateA**	69.32	60.76	77.14	32086	<2e-05	***
**SubstrateB**	53.72	46.35	60.4	48000	<2e-05	***
**SubstrateC**	49.36	42.26	56.36	48000	<2e-05	***
**SubstrateD**	49.98	41.88	57.78	38418	<2e-05	***
**SubstrateE**	46.45	38.29	54.2	25337	<2e-05	***
**SubstrateF**	50.74	42.83	58.14	30504	<2e-05	***
**G-structure:**						
**Distribution**	54.4	4.97E-17	163.9	45984		
**Log.body.mass**	0.6708	1.17E-16	3.81	1042		

Signif. codes: 0 '***' 0.001 '**' 0.01 '*' 0.05 '.' 0.1 ' ' 1

Table S.9. Model selection output of MCMCglmm Dorsal ~ -1+ Substrate + Ventral + Altitude + Distribution + Log body mass + Polymorphic + Day cycle. Only mostly supported models (DIC <5) are reported for graphical interpretation. Model = Model number from the MCMCglmm chain, Alt=Altitude, Day.cyc=Day cycle, Log.bdy.mss=Log body mass, Ply=Polymorphic, Sbs=Substrate, Vnt=Ventral.

Model	Alt	Day.cyc	Log.bdy.mss	Ply	Sbs	Vnt	df	logLik	DIC	delta	weight
36	+	+			+		14	-385.718	785.3	0	0.266
44	+	+	-0.8869		+		15	-385.714	786.3	0.94	0.166
52	+	+		+	+		15	-385.845	786.6	1.24	0.143
100	+	+			+	-0.01796	15	-386.236	787.3	1.99	0.098
60	+	+	-0.8131	+	+		16	-385.969	787.7	2.39	0.081
108	+	+	-0.8716		+	-0.00334	16	-386.293	788.4	3.11	0.056
116	+	+		+	+	-0.01046	16	-386.383	788.6	3.32	0.051
51		+		+	+		11	-388.938	788.8	3.43	0.048
35		+			+		10	-389.647	789.2	3.87	0.038
124	+	+	-0.8246	+	+	0.002712	17	-386.522	789.9	4.54	0.027
34	+				+		11	-389.598	790.1	4.77	0.025

Table S.10 Cumulative Akaike weights of the parameters employed in the dorsal full models. Sbs=Substrate, Day.cyc=Day cycl, Alt=Altitude, Ply=Polymorphic, Log.bdy.mss=Log body mass, Vnt=Ventral, Dst=Distribution.

	Sbs	Day.cyc	Alt	Ply	Log.bdy.mss	Vnt	Dst
**Sum of weights:**	1	0.93	0.85	0.37	0.35	0.26	0.01
**N containing models:**	64	64	64	64	64	64	64

Table S.11. Output of the MCMCglmm model: Dorsal ~ Substrate + Day cycle + Altitude. DIC = 785.32. Reference level: Substrate A + Altitude All + Day cycle Both. Substrate A = arid substrates, Substrate B = arid substrates + grass +/ forest grounds, Substrate C = generalists, Substrate D = grass + forest grounds, Substrate E = high vegetation, Substrate F = forest grounds.

	post.mean	l-95% CI	u-95% CI	eff.samp	pMCMC	
**(Intercept)**	45.72	42.38	49.07	48000	< 2e-05	***
**SubstrateB**	-5.42	-8.99	-1.81	48000	0.00417	**
**SubstrateC**	-8.28	-11.74	-4.74	49056	< 2e-05	***
**SubstrateD**	-9.41	-13.38	-5.46	48000	< 2e-05	***
**SubstrateE**	-4.61	-8.49	-0.70	48000	0.02158	*
**SubstrateF**	-9.34	-13.03	-5.77	48000	< 2e-05	***
**AltitudeHigh**	-2.18	-5.06	0.77	48000	0.1415	
**AltitudeLow**	3.11	0.21	6.16	46905	0.04054	*
**AltitudeLow-Medium**	-1.02	-4.05	2.08	49020	0.51179	
**AltitudeMedium-High**	-3.69	-7.93	0.65	48000	0.09183	.
**Day.cycleDiurnal**	3.30	0.69	5.87	48000	0.01317	*
**Day.cycleNocturnal**	0.40	-2.07	2.83	46239	0.74638	
**Day.cycleUnknown**	-4.36	-11.37	2.31	48000	0.20704	

Signif. codes: 0 ‘***’ 0.001 ‘**’ 0.01 ‘*’ 0.05 ‘.’ 0.1 ‘ ’ 1

Table S.12. Reconstructed posterior means [lower, upper 95% credible intervals] of the dorsal brightness from the output of the MCMCglmm model in Table S.10. Substrate A = arid substrates, Substrate B = arid substrates + grass +/ forest grounds, Substrate C = generalists, Substrate D = grass + forest grounds, Substrate E = high vegetation, Substrate F = forest grounds.

	Altitude					
**Day cycle: Diurnal**	**Low**	**Low-Medium**	**Medium-High**	**High**	**All**	**Substrate Mean**
**SubstrateA**	52.13 [43.28,61.09]	48.00 [39.02,57.01]	45.33 [35.14,55.59]	46.84 [38.00,55.71]	49.02 [43.07,54.94]	48.27 [39.70,56.87]
**SubstrateB**	46.71 [34.29,59.28]	42.59 [30.03,55.20]	39.92 [26.16,53.78]	41.42 [29.02,53.90]	43.60 [34.08,53.13]	42.85 [30.72,55.06]
**SubstrateC**	43.85 [31.53,56.35]	39.73 [27.28,52.27]	37.06 [23.40,50.84]	38.56 [26.26,50.96]	40.74 [31.33,50.19]	39.99 [27.96,52.12]
**SubstrateD**	42.72 [29.89,55.64]	38.59 [25.64,51.56]	35.92 [21.76,50.13]	37.43 [24.62,50.25]	39.61 [29.69,49.48]	38.85 [26.32,51.41]
**SubstrateE**	47.52 [34.78,60.40]	43.40 [30.53,56.32]	40.73 [26.65,54.89]	42.24 [29.51,55.01]	44.41 [34.58,54.24]	43.66 [31.21,56.17]
**SubstrateF**	42.79 [30.25,55.33]	38.66 [25.99,51.25]	35.99 [22.11,49.82]	37.50 [24.98,49.94]	39.68 [30.04,49.17]	38.92 [26.67,51.10]
**Altitude Mean**	45.95 [34.00,58.01]	41.83 [29.75,53.93]	39.16 [25.87,52.51]	40.67 [28.73,52.63]	42.84 [33.80,51.86]	
**Day cycle Mean**	42.09 [30.43,53.79]					
**Day cycle: Nocturnal**						
**SubstrateA**	49.23 [40.52,58.06]	45.10 [36.27,53.98]	42.43 [32.39,52.55]	43.94 [35.25,52.68]	46.12 [40.32,51.91]	45.36 [36.95,53.84]
**SubstrateB**	43.81 [31.54,56.25]	39.68 [27.28,52.17]	37.01 [23.40,50.74]	38.52 [26.27,50.87]	40.70 [31.33,50.10]	39.94 [27.96,52.03]
**SubstrateC**	40.95 [28.78,53.32]	36.82 [24.52,49.24]	34.15 [20.65,47.81]	35.66 [23.51,47.93]	37.84 [28.57,47.16]	37.08 [25.21,49.09]
**SubstrateD**	39.81 [27.14,52.60]	35.69 [22.88,48.53]	33.02 [19.01,47.10]	34.52 [21.87,47.22]	36.70 [26.93,46.45]	35.95 [23.57,48.38]
**SubstrateE**	44.62 [32.03,57.36]	40.49 [27.78,53.28]	37.82 [23.90,51.86]	39.33 [26.76,51.98]	41.51 [31.82,51.21]	40.76 [28.46,53.14]
**SubstrateF**	39.89 [27.50,52.29]	35.76 [23.24,48.22]	33.09 [19.36,46.79]	34.60 [22.22,46.91]	36.78 [27.29,46.14]	36.02 [23.92,48.07]
**Altitude Mean**	43.05 [31.25,54.98]	38.92 [27.00,50.90]	36.25 [23.12,49.47]	37.76 [25.98,49.60]	39.94 [31.04,48.83]	
**Day cycle Mean**	39.19 [27.68,50.76]					
**Day cycle: Both**						
**SubstrateA**	48.83 [42.59,55.23]	44.70 [38.34,51.15]	42.03 [34.46,49.72]	43.54 [37.32,49.84]	45.72 [42.38,49.07]	44.96 [39.02,51.00]
**SubstrateB**	43.41 [33.61,53.42]	39.28 [29.35,49.34]	36.61 [25.47,47.91]	38.12 [28.33,48.03]	40.30 [33.40,47.26]	39.54 [30.03,49.19]
**SubstrateC**	40.55 [30.85,50.48]	36.42 [26.59,46.40]	33.75 [22.71,44.97]	35.26 [25.58,45.10]	37.44 [30.64,44.33]	36.68 [27.27,46.26]
**SubstrateD**	39.41 [29.21,49.77]	35.28 [24.95,45.69]	32.61 [21.07,44.26]	34.12 [23.94,44.39]	36.30 [29.00,43.61]	35.55 [25.63,45.54]
**SubstrateE**	44.22 [34.10,54.53]	40.09 [29.84,50.45]	37.42 [25.97,49.02]	38.93 [28.83,49.14]	41.11 [33.89,48.37]	40.36 [30.53,50.30]
**SubstrateF**	39.48 [29.56,49.46]	35.36 [25.31,45.38]	32.69 [21.43,43.95]	34.20 [24.29,44.08]	36.37 [29.36,43.30]	35.62 [25.99,45.23]
**Altitude Mean**	42.65 [33.32,52.15]	38.52 [29.06,48.07]	35.85 [25.19,46.64]	37.36 [28.05,46.76]	39.54 [33.11,45.99]	
**Day cycle Mean**	38.79 [29.75,47.92]					
**Day cycle: Unknown**						
**SubstrateA**	44.47 [31.22,57.53]	40.34 [26.97,53.45]	37.67 [23.09,52.03]	39.18 [25.95,52.15]	41.36 [31.01,51.38]	40.60 [27.55,53.31]
**SubstrateB**	39.05 [22.24,55.72]	34.92 [17.98,51.65]	32.25 [14.10,50.22]	33.76 [16.96,50.34]	35.94 [22.03,49.57]	35.19 [18.66,51.50]
**SubstrateC**	36.19 [19.48,52.79]	32.06 [15.22,48.71]	29.39 [11.34,47.28]	30.90 [14.21,47.40]	33.08 [19.27,46.63]	32.33 [15.90,48.56]
**SubstrateD**	35.05 [17.84,52.08]	30.93 [13.58,48.00]	28.26 [9.70,46.57]	29.76 [12.56,46.69]	31.94 [17.63,45.92]	31.19 [14.26,47.85]
**SubstrateE**	39.86 [22.73,56.84]	35.73 [18.47,52.76]	33.06 [14.60,51.33]	34.57 [17.46,51.45]	36.75 [22.52,50.68]	36.00 [19.16,52.61]
**SubstrateF**	35.13 [18.19,51.77]	31.00 [13.94,47.69]	28.33 [10.06,46.26]	29.84 [12.92,46.38]	32.02 [17.98,45.61]	31.26 [14.62,47.54]

**Altitude Mean**	38.29 [21.95,54.46]	34.16 [17.69,50.38]	31.49 [13.82,48.95]	33.00 [16.68,49.07]	35.18 [21.74,48.30]
**Day cycle Mean**	34.43 [18.37,50.23]				

Table S.13. Output of the graphical MCMCglmm model Dorsal ~ -1 + Substrate | Altitude + Day cycle. DIC= 794.18. Substrate A = arid substrates, Substrate B = arid substrates + grass +/ forest grounds, Substrate C = generalists, Substrate D = grass + forest grounds, Substrate E = high vegetation, Substrate F = forest grounds.

	**post.mean**	**l-95% CI**	**u-95% CI**	**eff.samp**	**pMCMC**	
**SubstrateA**	46.33	42.4	50.27	44474	< 2e-05	***
**SubstrateB**	41.14	37.72	44.37	24422	< 2e-05	***
**SubstrateC**	38.11	34.65	41.32	37504	< 2e-05	***
**SubstrateD**	36.58	32.95	40.43	32992	4.17e-05	***
**SubstrateE**	40.33	37.1	43.85	48000	< 2e-05	***
**SubstrateF**	36.96	33.53	40.29	35216	< 2e-05	***
**G-structure:**						
**Day.cycle**	5.167	1.55e-17	19.69	35603		
**Altitude**	2.795	1.73e-17	13.19	16964		

Signif. codes: 0 ‘***’ 0.001 ‘**’ 0.01 ‘*’ 0.05 ‘.’ 0.1 ‘ ’ 1

Table S.14. Model selection output of MCMCglmm Head ~ -1+ Substrate + Ventral + Altitude + Distribution + Log body mass + Polymorphic + Day cycle. Only mostly supported models (DIC <5) are reported for graphical interpretation. Model = Model number from the MCMCglmm chain, Alt=Altitude, Day.cyc=Day cycle, Log.bdy.mss=Log body mass, Ply=Polymorphic, Sbs=Substrate, Vnt=Ventral.

Model	Alt	Day.cyc	Log.bdy.mss	Ply	Sbs	Vnt	df	logLik	DIC	delta	weight
44	+	+	-1.878		+		15	-419.017	852.9	0	0.173
36	+	+			+		14	-419.985	853.8	0.91	0.11
108	+	+	-2.18		+	0.06547	16	-419.122	854.1	1.15	0.097
60	+	+	-1.79	+	+		16	-419.408	854.6	1.72	0.073
42	+		-1.697		+		12	-421.464	854.9	1.94	0.066
34	+				+		11	-422.122	855.2	2.24	0.057
52	+	+		+	+		15	-420.21	855.3	2.36	0.053
124	+	+	-2.116	+	+	0.07216	17	-419.377	855.6	2.66	0.046
100	+	+			+	0.02926	15	-420.438	855.8	2.83	0.042
35		+			+		10	-422.981	855.9	2.97	0.039
51		+		+	+		11	-422.593	856.1	3.16	0.036
106	+		-1.91		+	0.04473	13	-421.788	856.5	3.58	0.029
58	+		-1.632	+	+		13	-421.875	856.6	3.69	0.027
50	+			+	+		12	-422.411	856.7	3.81	0.026
116	+	+		+	+	0.03885	16	-420.587	857.1	4.14	0.022
98	+				+	0.0129	12	-422.654	857.2	4.29	0.02
43		+	-0.8665		+		11	-423.185	857.3	4.34	0.02
59		+	-0.7741	+	+		12	-422.856	857.6	4.7	0.017
33					+		7	-425.393	857.7	4.82	0.016
115		+		+	+	0.04281	12	-422.947	857.8	4.85	0.015
99		+			+	0.0234	11	-423.457	857.8	4.92	0.015

Table S.15. Cumulative Akaike weights of the parameters employed in the head full models. Sbs=Substrate, Alt=Altitude, Day.cyc=Day cycle, Log.bdy.mss=Log body mass, Ply=Polymorphic, Vnt=Ventral, Dst=Distribution.

	Sbs	Alt	Day.cyc	Log.bdy.mss	Ply	Vnt	Dst
**Sum of weights:**	1	0.79	0.71	0.55	0.35	0.32	0.02
**N containing models:**	64	64	64	64	64	64	64

Table S.16. Output of the MCMCglmm model: Head ~ Substrate + Altitude. DIC = 855.12. Reference level: Substrate A + Altitude All. Substrate A = arid substrates, Substrate B = arid substrates + grass +/ forest grounds, Substrate C = generalists, Substrate D = grass + forest grounds, Substrate E = high vegetation, Substrate F = forest grounds.

	post.mean	l-95% CI	u-95% CI	eff.samp	pMCMC	
**(Intercept)**	50.67	46.97	54.63	48000	< 2e-05	***
**SubstrateB**	-5.91	-10.45	-1.29	43681	0.011125	*
**SubstrateC**	-10.95	-15.65	-6.29	48000	< 2e-05	***
**SubstrateD**	-13.87	-19.11	-8.57	48656	< 2e-05	***
**SubstrateE**	-9.44	-14.69	-4.37	48000	0.000458	***
**SubstrateF**	-14.11	-18.84	-9.18	48000	< 2e-05	***
**AltitudeHigh**	-2.65	-6.31	0.97	48208	0.152167	
**AltitudeLow**	3.16	-0.76	7.09	48000	0.118167	
**AltitudeLow-Medium**	-2.45	-6.59	1.55	48000	0.233167	
**AltitudeMedium-High**	-5.43	-11.21	0.21	47382	0.062875	.

Signif. codes: 0 '***' 0.001 '**' 0.01 '*' 0.05 '.' 0.1 ' ' 1

Table S.17. Reconstructed posterior means [lower, upper 95% credible intervals] of the head brightness from the output of the MCMCglmm model in Table S.15. Substrate A = arid substrates, Substrate B = arid substrates + grass +/ forest grounds, Substrate C = generalists, Substrate D = grass + forest grounds, Substrate E = high vegetation, Substrate F = forest grounds.

			**Altitude**			
	**Low**	**Low-Medium**	**Medium-High**	**High**	**All**	**Substrate Mean**
**SubstrateA**	53.82 [46.20,61.73]	48.22 [40.38,56.19]	45.23 [35.75,54.84]	48.01 [40.66,55.60]	50.67 [46.97,54.63]	49.19 [41.99,56.60]
**SubstrateB**	47.92 [35.76,60.43]	42.31 [29.93,54.90]	39.33 [25.31,53.55]	42.11 [30.21,54.31]	44.76 [36.52,53.64]	43.29 [31.54,55.31]
**SubstrateC**	42.87 [30.56,55.43]	37.26 [24.73,49.89]	34.28 [20.11,48.55]	37.06 [25.01,49.31]	39.72 [31.32,48.34]	38.24 [26.34,50.31]
**SubstrateD**	39.95 [27.09,53.15]	34.35 [21.26,47.61]	31.36 [16.64,46.27]	34.14 [21.55,47.03]	36.80 [27.86,46.06]	35.32 [22.88,48.03]
**SubstrateE**	44.38 [31.51,57.35]	38.77 [25.68,51.81]	35.79 [21.06,50.47]	38.57 [25.97,51.23]	41.22 [32.28,50.26]	39.75 [27.30,52.23]
**SubstrateF**	39.72 [27.36,52.55]	34.11 [21.54,47.01]	31.13 [16.91,45.66]	33.91 [21.82,46.43]	36.56 [28.13,45.46]	35.08 [23.15,47.42]
**Altitude Mean**	44.78 [33.08,56.77]	39.17 [27.25,51.24]	36.19 [22.63,49.89]	38.97 [27.54,50.65]	41.62 [33.84,49.68]	

Table S.18. Output of the graphical MCMCglmm model Head ~ -1 + Substrate | Altitude. DIC= 857.65. Substrate A = arid substrates, Substrate B = arid substrates + grass +/ forest grounds, Substrate C = generalists, Substrate D = grass + forest grounds, Substrate E = high vegetation, Substrate F = forest grounds.

	post.mean	l-95% CI	u-95% CI	eff.samp	pMCMC	
SubstrateA	50.4	46.36	54.57	37889	<2e-05	***
SubstrateB	44.33	41.53	47.19	37502	<2e-05	***
SubstrateC	39.18	36.19	42.3	43177	<2e-05	***
SubstrateD	35.44	31.6	39.17	48000	<2e-05	***
SubstrateE	39.65	36.4	42.92	48000	<2e-05	***
SubstrateF	36.3	33.09	39.56	36467	<2e-05	***
G-structure:						
Altitude	1.696	1.29E-17	8.672	15072		

Signif. codes: 0 ‘***’ 0.001 ‘**’ 0.01 ‘*’ 0.05 ‘.’ 0.1 ‘ ’ 1

Table S.19. Model selection output of MCMCglmm Pattern ~ -1+ Substrate + Ventral + Altitude + Distribution + Log body mass + Polymorphic + Day cycle. Only mostly supported models (DIC <5) are reported for graphical interpretation. Model = Model number from the MCMCglmm chain, Day.cyc=Day cycle, Dst=Distribution, Log.bdy.mss=Log body mass, Ply=Polymorphic, Sbs=Substrate, Vnt=Ventral.

**Model**	**Day.cyc**	**Dst**	**Log.bdy.mss**	**Ply**	**Sbs**	**Vnt**	**df**	**logLik**	**DIC**	**delta**	**weight**
37		+			+		12	-446.586	905.1	0	0.128
35	+				+		10	-447.922	905.7	0.65	0.092
101		+			+	0.1278	13	-446.43	905.8	0.69	0.09
39	+	+			+		15	-445.489	905.8	0.74	0.088
103	+	+			+	0.1259	16	-445.322	906.5	1.44	0.062
45		+	0.5804		+		13	-447.089	907.1	1.99	0.047
53		+		+	+		13	-447.172	907.2	2.11	0.044
33					+		7	-450.14	907.2	2.11	0.044
99	+				+	0.05956	11	-448.287	907.5	2.39	0.039
43	+		0.6885		+		11	-448.363	907.6	2.56	0.035
51	+			+	+		11	-448.47	907.8	2.76	0.032
117		+		+	+	0.1308	14	-446.994	907.9	2.76	0.032
109		+	0.1715		+	0.1244	14	-447	907.9	2.77	0.032
47	+	+	0.1508		+		16	-446.034	907.9	2.84	0.031
55	+	+		+	+		16	-446.061	908	2.86	0.03
111	+	+	-0.3014		+	0.1305	17	-445.919	908.6	3.53	0.022
119	+	+		+	+	0.1278	17	-445.91	908.6	3.56	0.022
41			0.7492		+		8	-450.544	909	3.94	0.018
61		+	0.5627	+	+		14	-447.681	909.2	4.09	0.016
97					+	0.02745	8	-450.635	909.2	4.12	0.016
49				+	+		8	-450.674	909.3	4.18	0.016
107	+		0.485		+	0.05093	12	-448.795	909.5	4.39	0.014
115	+			+	+	0.06393	12	-448.802	909.5	4.42	0.014
59	+		0.6973	+	+		12	-448.922	909.7	4.64	0.013
125		+	0.1753	+	+	0.1279	15	-447.549	910	4.88	0.011
63	+	+	0.1372	+	+		17	-446.652	910.1	4.98	0.011

Table S.20. Cumulative Akaike weights of the parameters employed in the pattern full models. Sbs=Substrate, Dst=Distribution, Day.cyc=Day cycle, Vnt=Ventral, Log.bdy.mss=Log body mass, Ply=Polymorphic, Alt=Altitude .

	Sbs	Dst	Day.cyc	Vnt	Log.bdy.mss	Ply	Alt
**Sum of weights:**	1	0.64	0.5	0.37	0.27	0.26	0.05
**N containing models:**	64	64	64	64	64	64	64

Table S.21. Output of the graphical MCMCglmm model Pattern ~ -1 + Substrate. DIC= 907.21. Substrate A = arid substrates, Substrate B = arid substrates + grass +/ forest grounds, Substrate C = generalists, Substrate D = grass + forest grounds, Substrate E = high vegetation, Substrate F = forest grounds.

	post.mean	l-95% CI	u-95% CI	eff.samp	pMCMC	
SubstrateA	43.09	37.37	48.55	48000	<2e-05	***
SubstrateB	32.61	28.85	36.22	48000	<2e-05	***
SubstrateC	32.06	27.66	36.33	48000	<2e-05	***
SubstrateD	26.85	21.5	31.99	48625	<2e-05	***
SubstrateE	36.06	31.35	40.9	52022	<2e-05	***
SubstrateF	26.44	22.13	30.81	48000	<2e-05	***

Signif. codes: 0 ‘***’ 0.001 ‘**’ 0.01 ‘*’ 0.05 ‘.’ 0.1 ‘ ’ 1

Table S.22. Frequency table of the categorical variables used in this study. For a classification description please refer to the main text. Substrate A = arid substrates, Substrate B = arid substrates + grass +/ forest grounds, Substrate C = generalists, Substrate D = grass + forest grounds, Substrate E = high vegetation, Substrate F = forest grounds. Altitude Low = x ≤ 500 m, Altitude Low-Medium = x ≤ 1000 m, Altitude Medium-High = 500 m < x ≤ 4000 m, Altitude High = 1000 < x ≤ 4000 m , Altitude All = all the range .

	**Category Frequencies**					
**Substrate**	*A*	*B*	*C*	*D*	*E*	*F*
(***n***)	13	30	25	15	21	22
**Altitude**	*Low*	*Low-Medium*	*Medium-High*	*High*	*All*	
(***n***)	15	14	7	20	70	
**Distribution**	*Tropical*	*Tropical-Subtropical*	*Subtropical*	*Subtropical-Temperate*	*Temperate*	*Temperate-Polar*
(***n***)	55	20	27	9	14	1
**Day Cycle**	*Diurnal*	*Nocturnal*	*Both*	*Unknown*		
(***n***)	31	59	33	3		
**Polymorphism**	*Yes*	*No*				
(***n***)	69	57				

Table S.23. Stayton's convergence metrics for the focal taxa derived from "convevol" analyses. Values in parentheses indicate significant values.

Convergent (Focal) Taxa	C1	C2	C3	C4	C5
Bitis parviocula, *Bitis peringueyi*, *Causus resimus, Causus defilippii*, *Daboia mauritanica*, *Eristicophis macmahoni*, *Pseudocerastes urarachnoides*, *Pseudocerastes persicus*, *Cerastes cerastes*, *Cerastes gasperettii*, *Echis pyramidum, Echis omanensis*, *Echis coloratus*, *Echis leucogaster*, *Crotalus ruber*, *Crotalus cerastes*	0.54 (0.00)	15.10 (0.00)	0.22 (0.00)	0.007 (0.00)	15 (0.00)

Notes: C1-C4 are distances and C5 is a frequency-based degree of convergence [3]. C1 denotes the proportion of maximum distance between two lineages that has been brought together by subsequent evolution, ranging from 0 (i.e. convergence is absent) to 1 (lineages are fully convergent). C2 indicates the absolute amount of evolution occurred during convergence. C3 represents the proportion of total evolutionary change between C2 and the lineages leading from the common ancestors of the convergent focal taxa to their tips (i.e. the focal taxa themselves). C4, similarly to C3, is still reporting the proportion of total evolutionary change, but between C2 and the entire clade defined by the common ancestor of the focal taxa. Finally, C5 is the number of focal taxa that are clustered within a convergent and limited area of the polymorphospace. For a graphical representation please refer to Figure S.14.

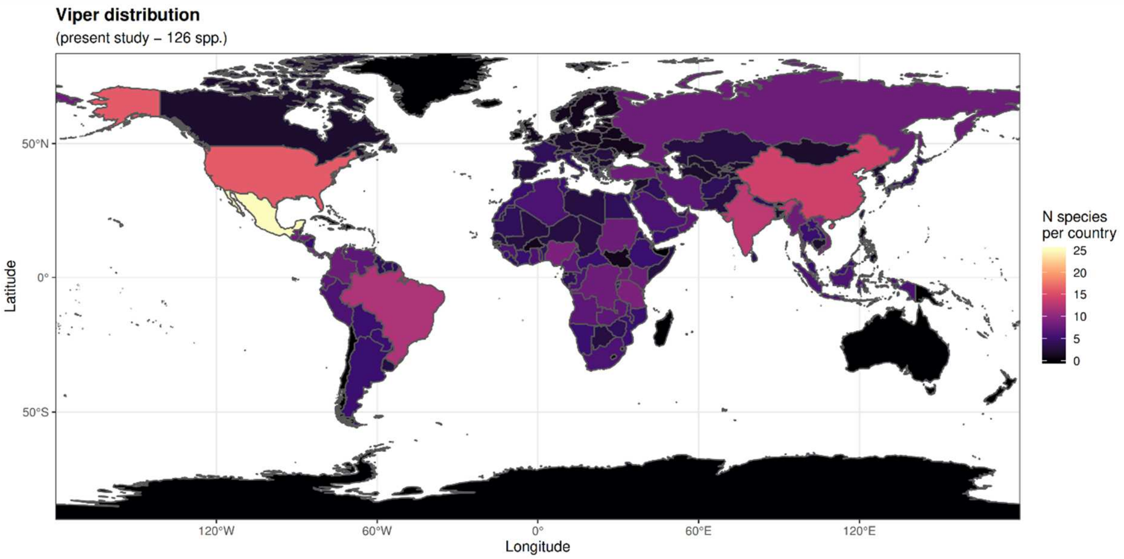

Figure S.1. Vipers distribution across the globe analyzed in this study: 126 species. Brighter colors denote higher species presence in any given country. Output created with "ggplot2" [4] using the country codes provided in "rnaturalearth" [5].

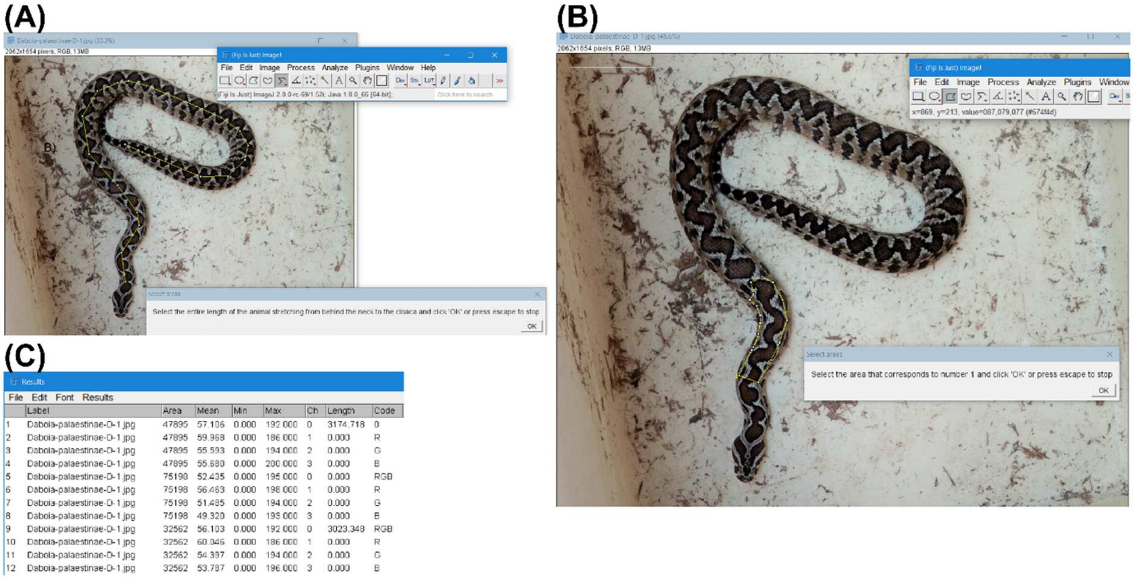

Figure S.2. Example of work-flow using our macro in ImageJ, for data brightness acquisition from the dorsum of an individual. Once the reference length is selected (**A**), the user is asked to select (if present) one of the three non-overlapping body areas (**B**). When the analysis is done, the macro returns a table that can be saved as .csv file **(C)** for further analyses. Credit: Jonathan Goldenberg, Palestine viper *(Daboia palaestinae*).

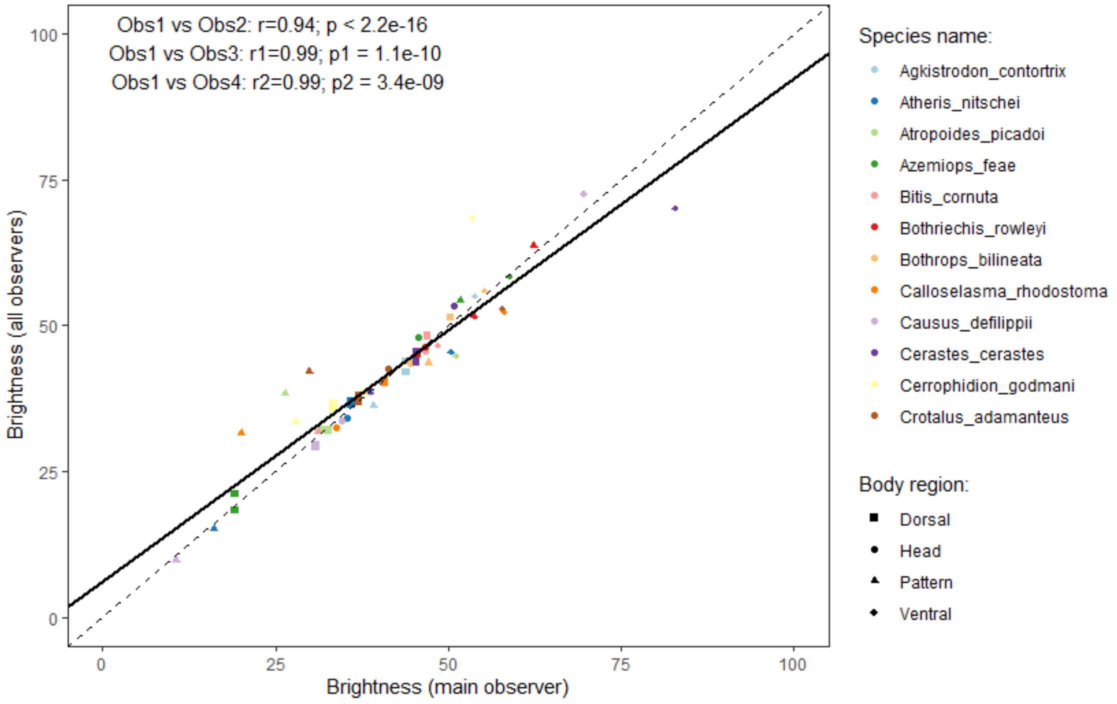

Figure S.3. Repeatability analysis. Pearson correlation analysis between observer (Obs) 1 and observer 2, 3, and 4 measured over 12 viper species from 388 images. Observer 2 and Observer 3 analyzed only the dorsal region. See main text for more details. Solid line represents the observed relationship between the observers. Dashed line: the ideal relationship with identical observations between observers.

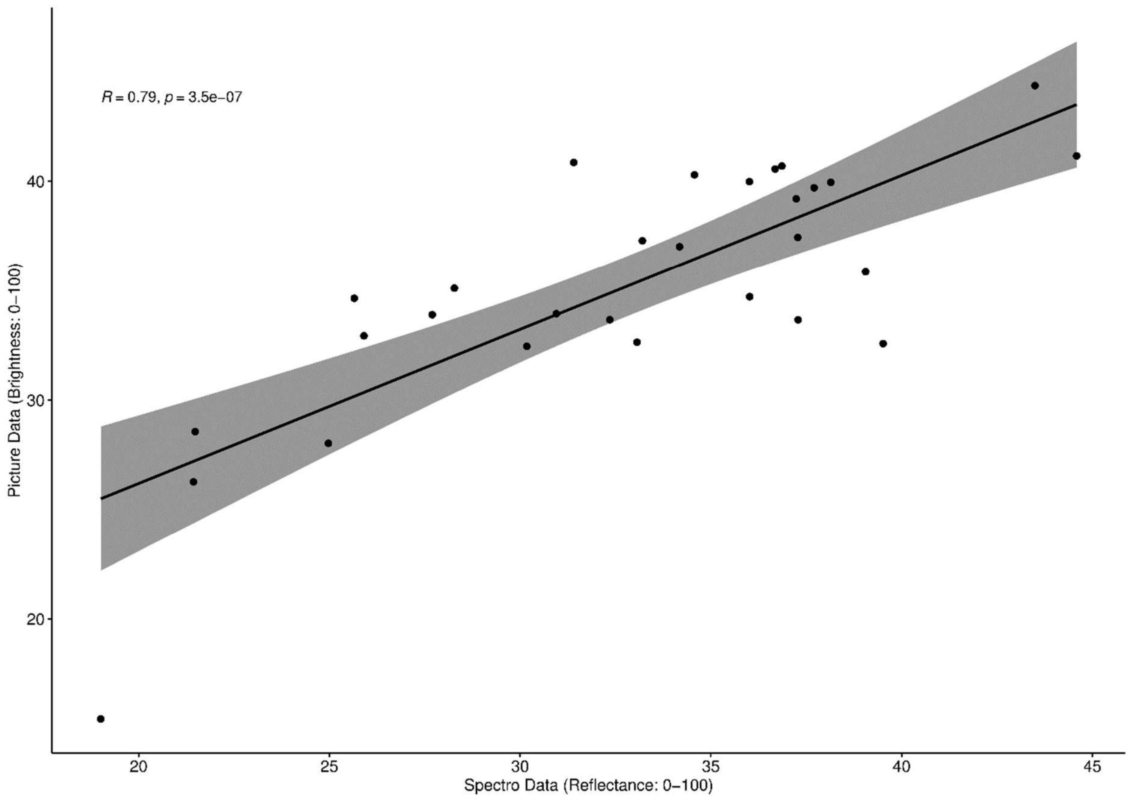

Figure S.4. Pearson correlation analysis between spectrophotometry and RGB brightness data. Results from 29 squamate species (see table S.2). Dots represent single species.

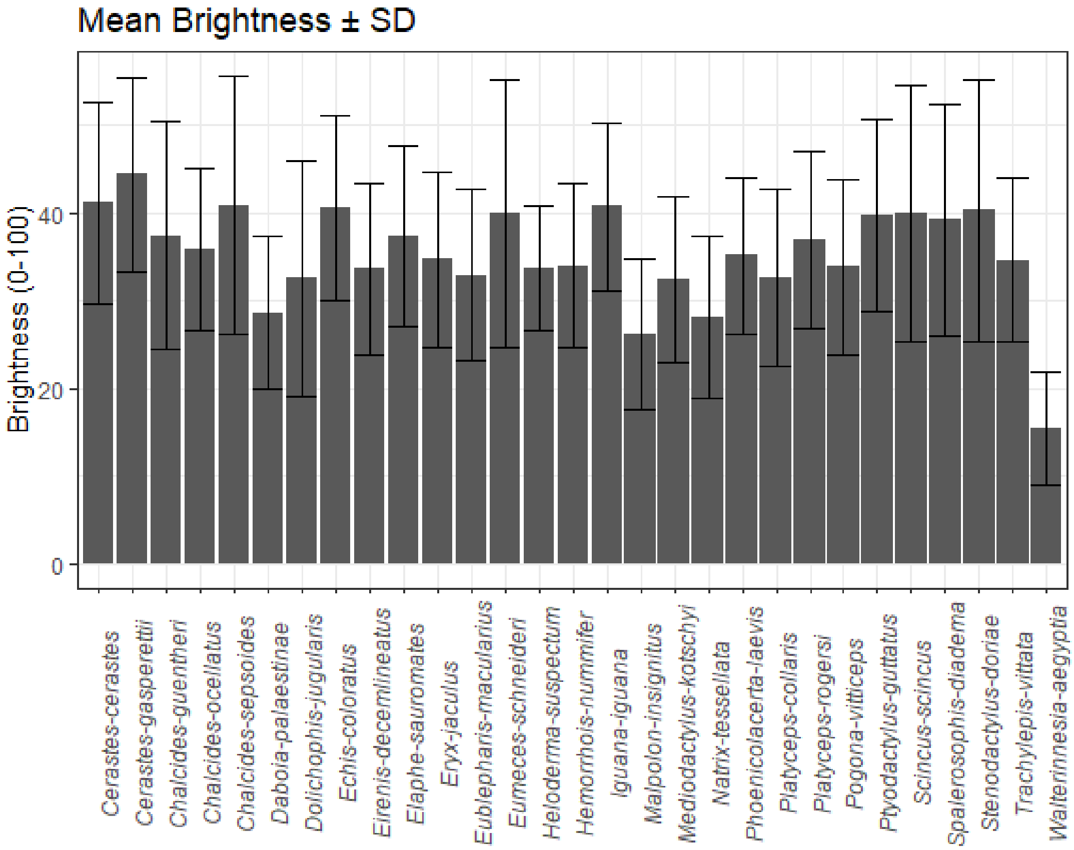

Figure S.5. Picture mean brightness ± standard deviation (SD) from the 29 squamate species used in the validation analysis between spectrophotometry and image brightness (Figure S.4). Table S.3 provides each of those mean brightness and SD values. Mean number of pictures per species = 9.81, SD = 1.41.

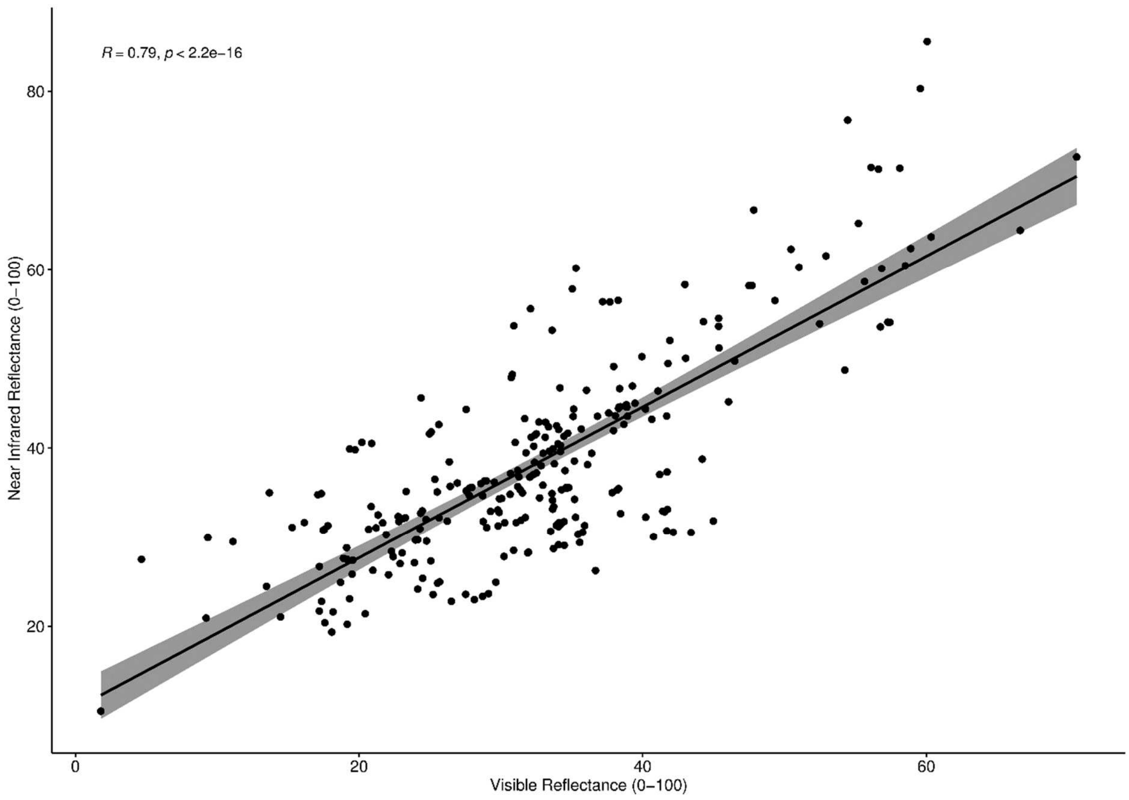

Figure S.6. Pearson correlation analysis between Visible (Vis: 380-740 nm) and Near Infrared (NIR: 740-1030 nm) spectral data from 29 squamate species (see table S.2). Dots represent single spectral measurements.

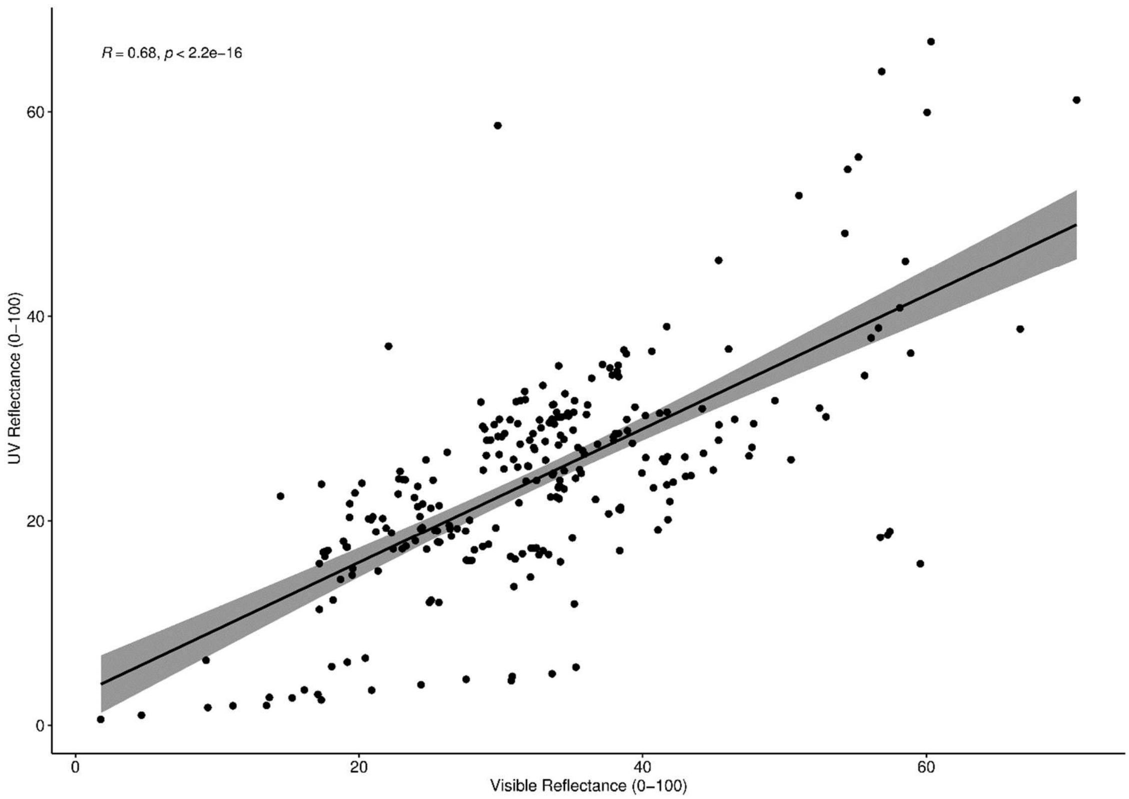

Figure S.7. Pearson correlation analysis between Visible (Vis: 380-740 nm) and Ultraviolet (UV: 299-380 nm) spectral data from 29 squamate species (see table S.2). Dots represent single spectral measurements.

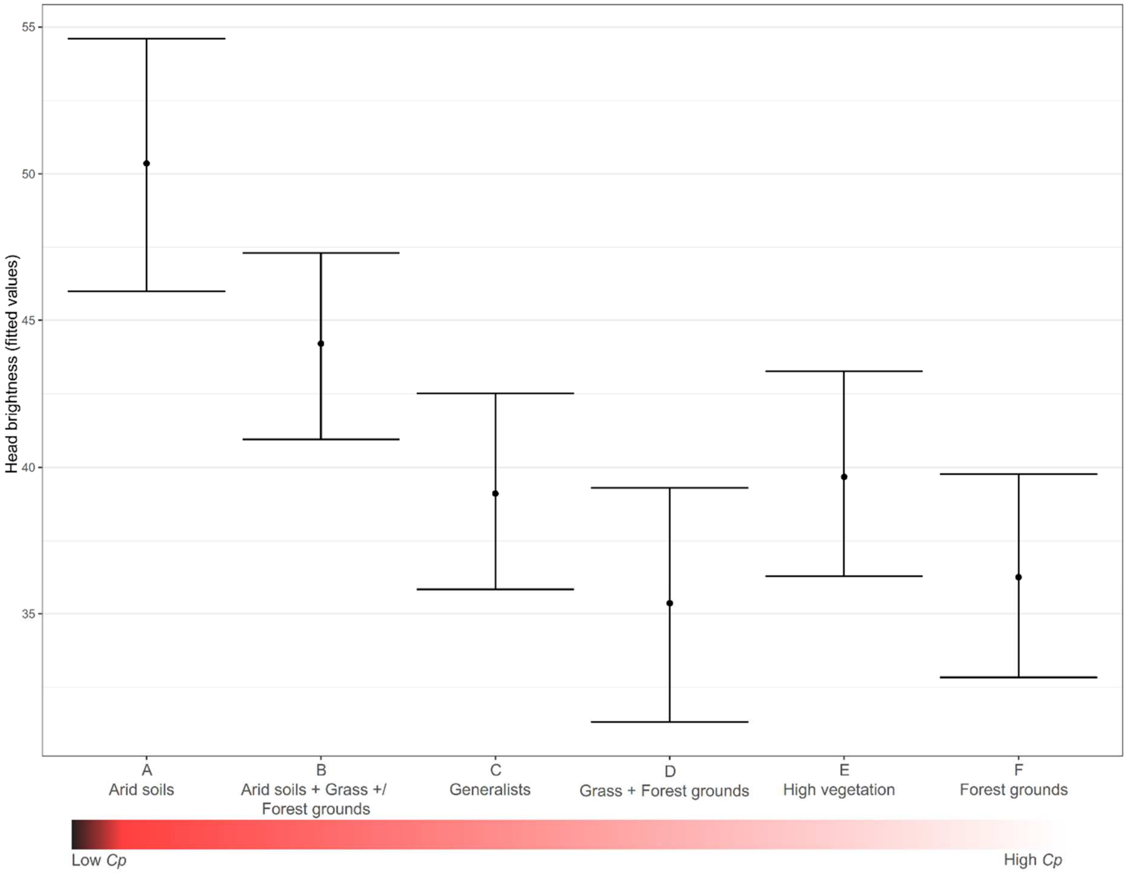

Figure S.8. MCMCglmm-predicted values of head brightness across different substrates. Bars represent 95% credible intervals. c_p = Specific Heat Capacity.

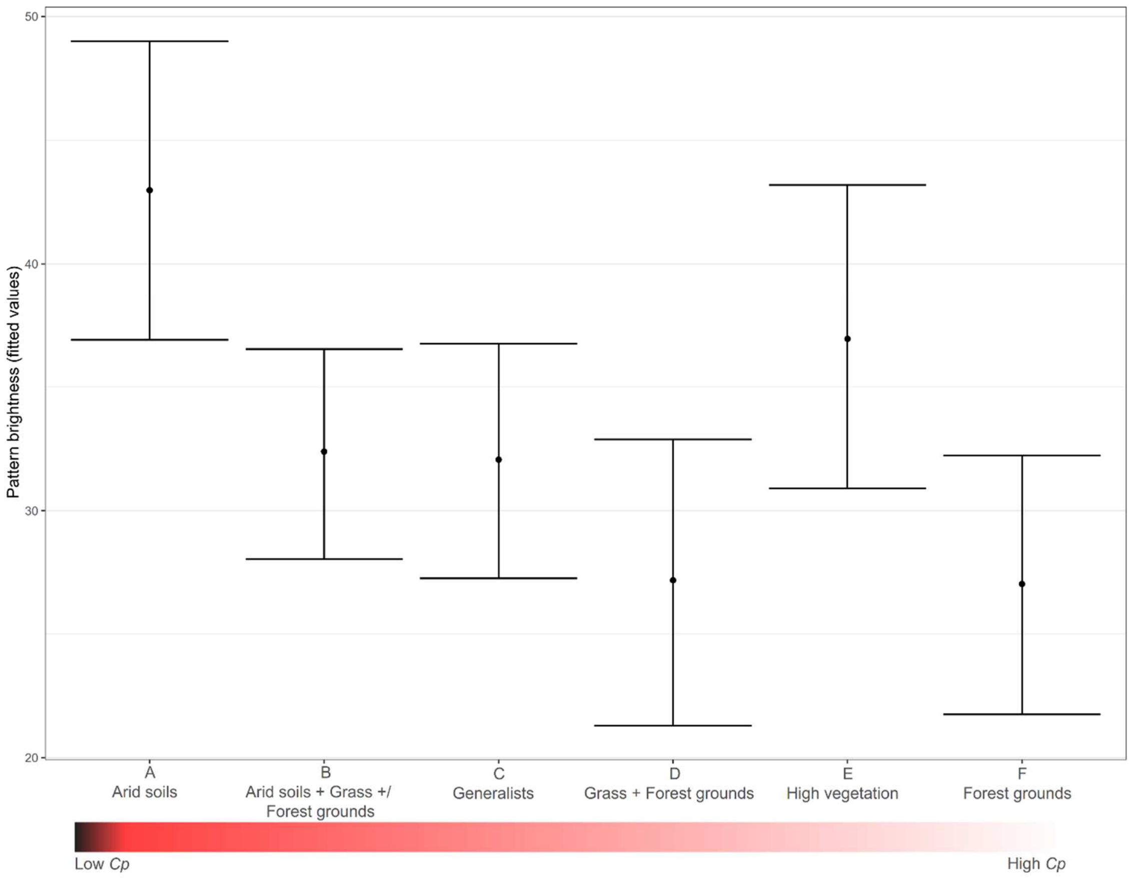

Figure S.9. MCMCglmm-predicted values of pattern brightness across different substrates. Bars represent 95% credible intervals. c_p = Specific Heat Capacity. Note that 6 species (*Atheris squamigera*, *Trimeresurus albolabris*, *T. erythrurus*, *T. medoensis*, *T. popeiorum*, *T. stejnegeri*) do not show pattern coloration, therefore we dropped them from this analysis.

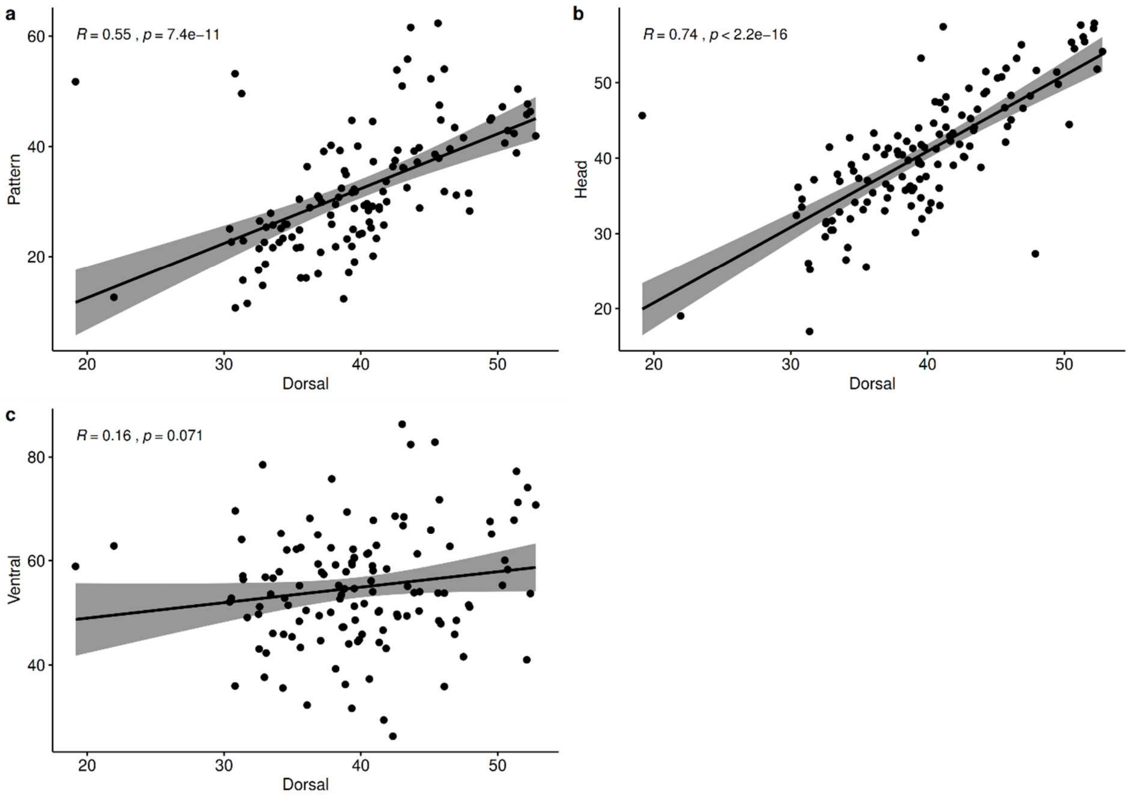

Figure S.10. Relationship between brightness levels of Dorsal, Head, Pattern and Ventral regions. Dots represent the studied species.

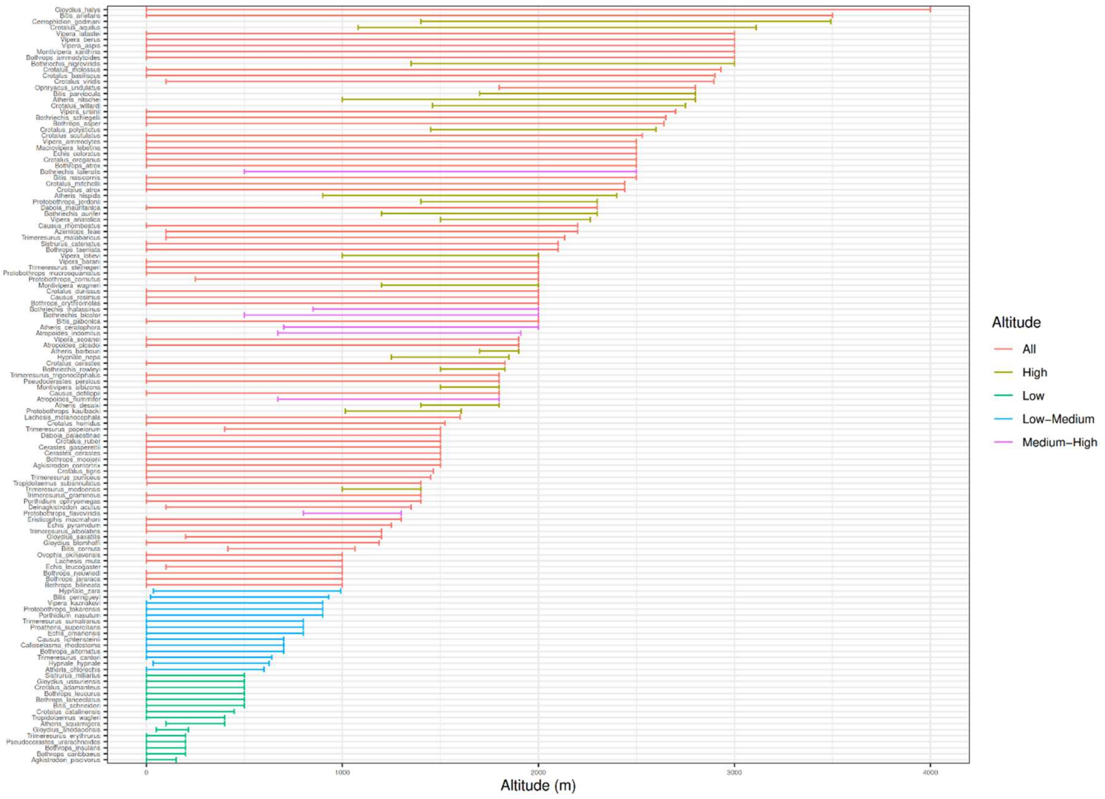

Figure S.11. Distribution of Altitude range across the 126 studied species. The classification follows the altitude thresholds presented in Körner et al. [6]. For a full description of category assignments, please refer to Methods in main text.

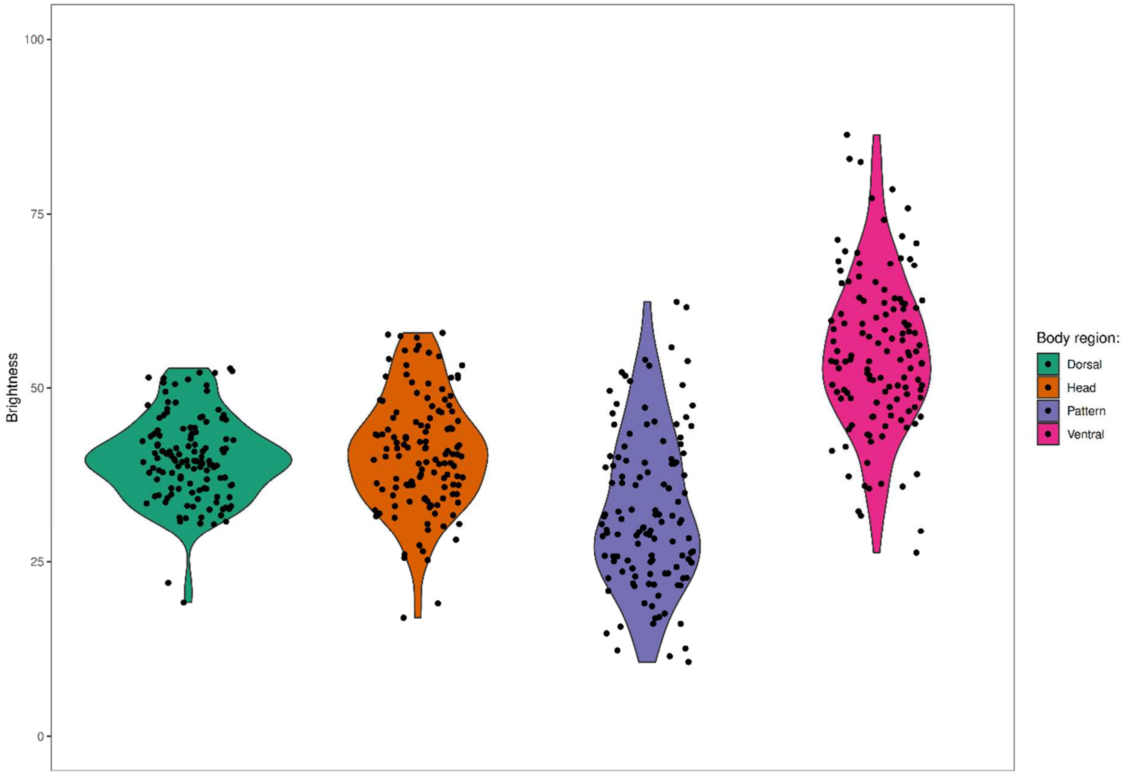

Figure S.12. Species mean brightness per each body region (ventrum, dorsum, head, pattern) from the 126 viper species. The ventral region is the brightest among the four regions.

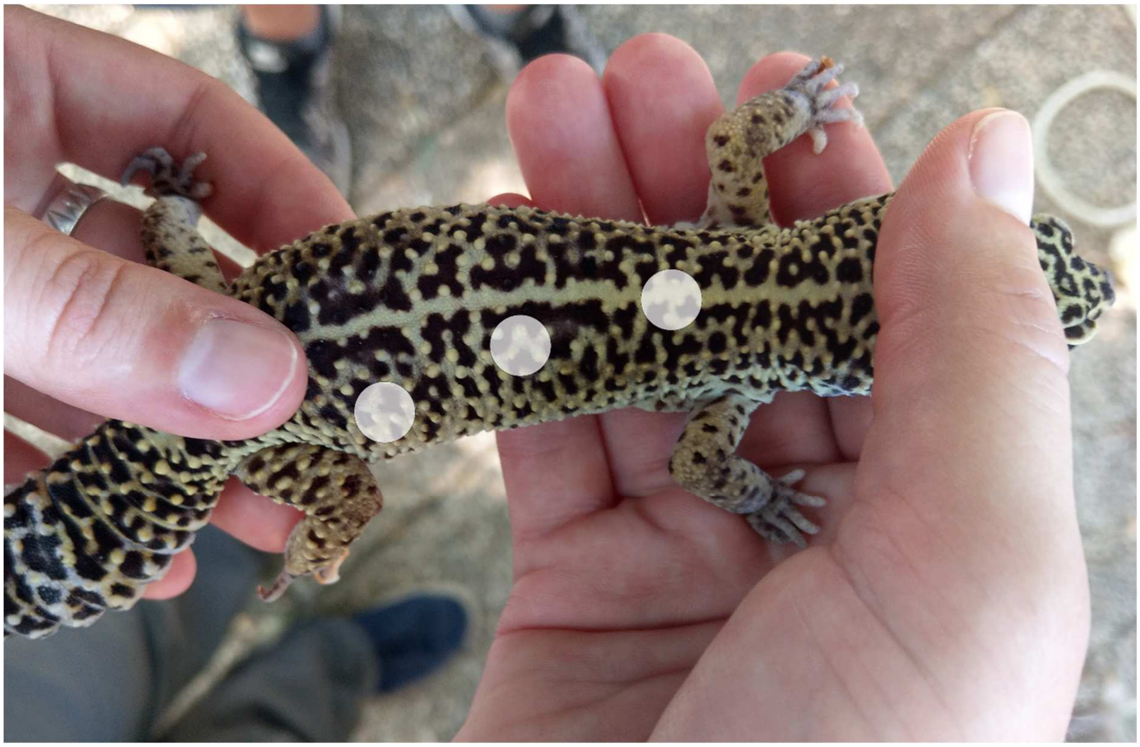

Figure S.13. The three spots on the trunk selected for the spectrophotometry analysis are shown in white circles. Here in example with the common leopard gecko (*Eublepharis macularius*). Credit: Jonathan Goldenberg.

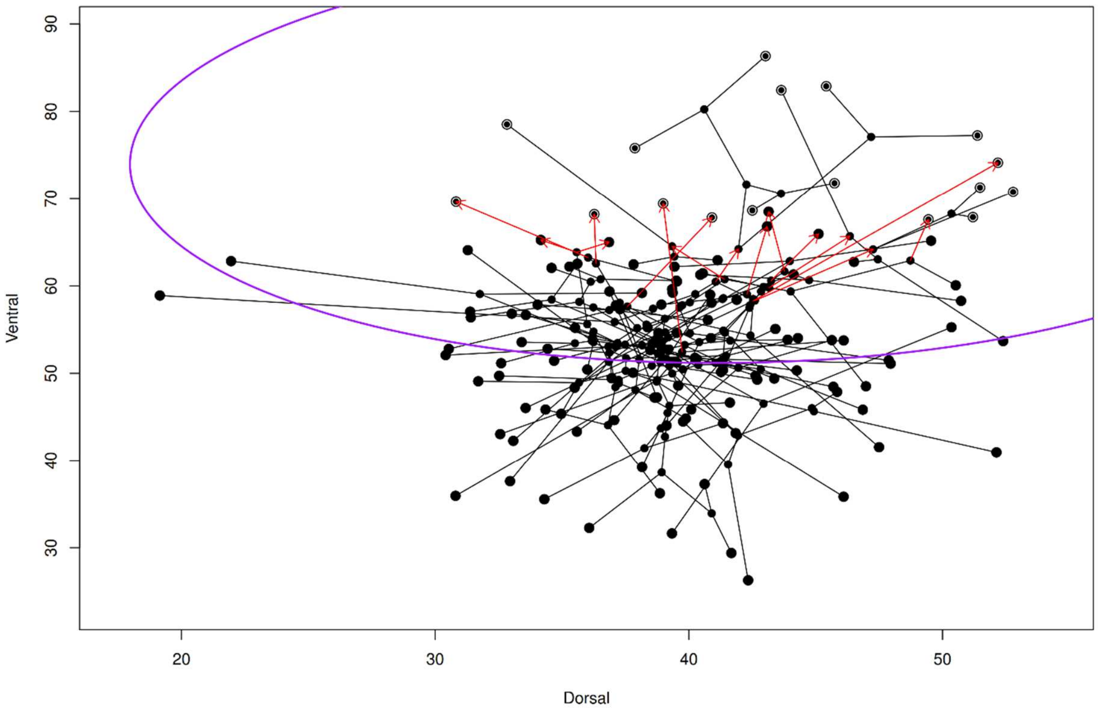

Figure S.14. Polymorphospace output from "convevol" defined by dorsal and ventral brightness on the 126 studied viper species showing convergent evolution for the focal species (for ventral brightness): *Bitis parviocula*, *Bitis peringueyi*, *Causus resimus, Causus defilippii*, *Daboia mauritanica*, *Eristicophis macmahoni*, *Pseudocerastes urarachnoides*, *Pseudocerastes persicus*, *Cerastes cerastes*, *Cerastes gasperettii*, *Echis pyramidum, Echis omanensis*, *Echis coloratus*, *Echis leucogaster*, *Crotalus ruber*, *Crotalus cerastes*. Full black dots denote non-focal species or nodes, and partially full dots denote focal species. Red arrows indicate the nodes/lineages that cross into the region of convergent species. The purple area defines the region where the focal taxa are present. The Stayton's metrics supporting this output are reported in Table S.23.

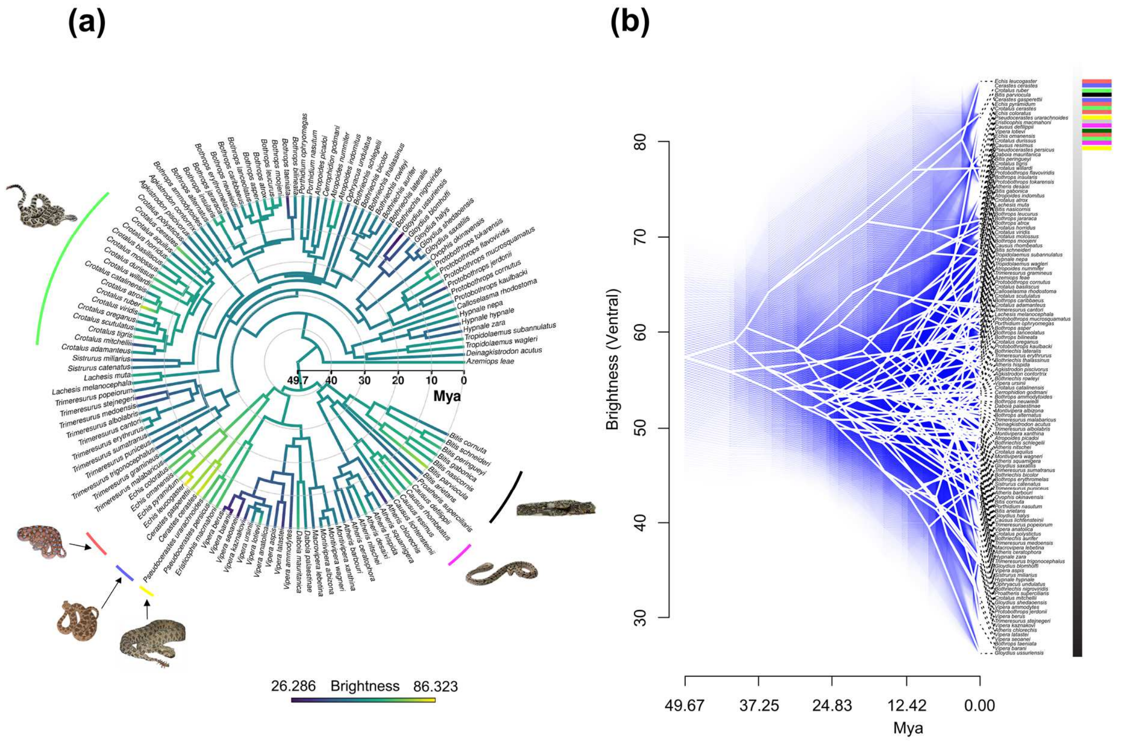

Figure S.15. a) Ancestral state estimation of the ventral brightness contrasted with a **b)** Phenogram with 95% confidence intervals projecting the phylogeny in a space defined by the ventral brightness and time showing that bright (and dark) ventral colors evolved independently over the vipers' evolutionary history; diverse colored-bars represent different genera. Only the first 16 brightest species have been colored for graphical interpretation. Credits: *Crotalus oreganus* (© Will Flaxington, CC BY-NC 3.0), *Echis coloratus* (© Matthieu Berroneau – with permission), *Cerastes cerastes* (© MinoZig, CC BY-NC 3.0.), *Pseudocerastes urarachnoides* (© Omid Mozaffari, CC-public domain), *Causus rhombeatus* (© Paul Venter, CC BY-NC 3.0.), *Bitis arietans* (© Jonathan Goldenberg).

REFERENCES (SUPPLEMENTARY)

1. Goldenberg, J., D'Alba, L. Bisschop, K., Vanthournout, B., Shawkey, M. (2020). "Replication Data for: Substrate thermal properties influence ventral brightness evolution in ectotherms"; https://doi.org/10.34894/FZ66NU, DataverseNL, V2.
2. Dehgan, B. (2014). *Public garden management: a global perspective* (Vol. 2). Xlibris Corporation.
3. Stayton, C. T. (2015). The definition, recognition, and interpretation of convergent evolution, and two new measures for quantifying and assessing the significance of convergence. *Evolution*, *69*(8), 2140-2153.
4. Wickham, H. (2016). *ggplot2: Elegant Graphics for Data Analysis*. Springer-Verlag New York. ISBN 978-3-319-24277-4
5. South, A. (2017). *rnaturalearth*: World Map Data from Natural Earth. R package version 0.1.0. https://CRAN.R-project.org/package=rnaturalearth
6. Körner, C., et al. (2005). Mountain Systems. Chapter 24 in: Millennium Ecosystem Assessment. Current State and Trends: Findings of the Condition and Trends Working Group. Ecosystems and Human Well-being, vol. 1.
