# Supplementary material for: Substrate thermal properties influence ventral brightness evolution in ectotherms
Source: Commun Biol. 2021 Jan 4;4:26. doi: 10.1038/s42003-020-01524-w (PMC7782800; doi:10.1038/s42003-020-01524-w)
Supplement: Supplementary file 3 — Reporting Summary [file 42003_2020_1524_MOESM3_ESM.pdf]

nature research

Corresponding author(s): Jonathan Goldenberg
jonathan.goldenberg@ugent.be

Last updated by author(s): Nov 4, 2020

Reporting Summary

Nature Research wishes to improve the reproducibility of the work that we publish. This form provides structure for consistency and transparency in reporting. For further information on Nature Research policies, see our Editorial Policies and the Editorial Policy Checklist.

Statistics

For all statistical analyses, confirm that the following items are present in the figure legend, table legend, main text, or Methods section.

n/a	Confirmed	
☐	☒	The exact sample size (n) for each experimental group/condition, given as a discrete number and unit of measurement
☐	☒	A statement on whether measurements were taken from distinct samples or whether the same sample was measured repeatedly
☐	☒	The statistical test(s) used AND whether they are one- or two-sided<br>*Only common tests should be described solely by name; describe more complex techniques in the Methods section.*
☐	☒	A description of all covariates tested
☐	☒	A description of any assumptions or corrections, such as tests of normality and adjustment for multiple comparisons
☐	☒	A full description of the statistical parameters including central tendency (e.g. means) or other basic estimates (e.g. regression coefficient) AND variation (e.g. standard deviation) or associated estimates of uncertainty (e.g. confidence intervals)
☐	☒	For null hypothesis testing, the test statistic (e.g. F, t, r) with confidence intervals, effect sizes, degrees of freedom and P value noted<br>*Give P values as exact values whenever suitable.*
☐	☒	For Bayesian analysis, information on the choice of priors and Markov chain Monte Carlo settings
☐	☒	For hierarchical and complex designs, identification of the appropriate level for tests and full reporting of outcomes
☐	☒	Estimates of effect sizes (e.g. Cohen's d, Pearson's r), indicating how they were calculated

Our web collection on statistics for biologists contains articles on many of the points above.

Software and code

Policy information about availability of computer code

Data collection	We collected 4161 images from peer-reviewed articles, field guides, Google Images, documentaries and our own data. The full dataset with all references is deposited at the provided repository. We collected spectrophotometry measurements on 29 squamate species at the Tel Aviv University's Garden for Zoological Research.
Data analysis	We analyzed all images in ImageJ 1.52i through a custom-made interactive plugin (macro) that guides the user to select the region of interests from which the researcher can obtain the brightness levels (.json file available at the provided repository). We conducted all analyses in R v.3.6.2 (R Core Team, 2019).

For manuscripts utilizing custom algorithms or software that are central to the research but not yet described in published literature, software must be made available to editors and reviewers. We strongly encourage code deposition in a community repository (e.g. GitHub). See the Nature Research guidelines for submitting code & software for further information.

Data

Policy information about availability of data

All manuscripts must include a data availability statement. This statement should provide the following information, where applicable:

- Accession codes, unique identifiers, or web links for publicly available datasets
- A list of figures that have associated raw data
- A description of any restrictions on data availability

_All R-scripts, datasets and the macro developed for ImageJ are available at: https://doi.org/10.34894/FZ66NU
_All supplementary tables and figures are provided in the Supplementary file

Field-specific reporting

Please select the one below that is the best fit for your research. If you are not sure, read the appropriate sections before making your selection.

☐ Life sciences ☐ Behavioural & social sciences ☒ Ecological, evolutionary & environmental sciences

For a reference copy of the document with all sections, see nature.com/documents/nr-reporting-summary-flat.pdf

Ecological, evolutionary & environmental sciences study design

All studies must disclose on these points even when the disclosure is negative.

Study description	The substrates on which ectotherms reside, and their integument brightness, vary in their heat transfer properties, thus, substrate use could influence skin brightness evolution. We predicted that species inhabiting hot, and highly radiative and superficially conductive substrates (i.e. low specific heat capacity (cp)) would express less melanic ventral integument than those on high cp substrates to better dissipate the ground heat. We analyzed 126 taxonomically unambiguous viper species from 31 genera, and we retrieved brightness levels on different body regions from 4161 images of these species from peer-reviewed articles, field guides, Google Images, documentaries and our own data. We noted the substrate type on which each species is thriving on. We also incorporated other environmental data that can significantly affect the amount of the sun's energy-rich radiation received by the animal and substrate (i.e. Altitude, Latitude), and morpho-behavioral information that can influence thermal heat transfers (i.e. Body mass, Polymorphism, Activity Pattern).
Research sample	Vipers (Viperidae Oppel, 1811) are a family of venomous snakes that evolved 50 Mya c.a.. Unlike other snakes, vipers use a sit-and-wait foraging behavior, and therefore their substrate type likely plays an important role in regulating their body temperature. To date, 365 viper species are distributed across the globe ranging from the tropics to the higher latitudes (> 60° N). The observed large diversity, coupled with their feeding strategy and a relatively long evolutionary history, makes this family an ideal study organism to investigate how ventral brightness evolved under divergent selective environments
Sampling strategy	Species selection was limited by the availability of ventral images. Our sample size is equally distributed across the phylogenetic tree and all levels have sufficient replicates for the convergence of our MCMCglmm models.
Data collection	One researcher collected all the images and constructed the dataset. Then, we jointly developed the macro in ImageJ that guides the user to select the region of interests from which any researcher can obtain the brightness levels . We further verified the repeatability of this methodology by assessing the observer variability. We also assessed the relationship between the visible (Vis), ultraviolet (UV), and near infrared (NIR) spectra. We finally analyzed all the data in R v.3.6.2 (R Core Team, 2019).
Timing and spatial scale	We collected images in January - June 2019. We acquired spectrophotometry measurements on the 15th of August 2019 and on the 1st of September 2019. We analyzed and processed the data from August 2019 - until February 2020.
Data exclusions	Six species (Atheris squamigera, Trimeresurus albolabris, T. erythrurus, T. medoensis, T. popeiorum, T. stejnegeri) do not show pattern coloration, therefore we dropped them from the analyses of this selected body region.
Reproducibility	To account for different lighting and setup conditions, we took the following steps: a) selected multiple (head (M=8.37, SD=2.09), dorsum (M=8.64, SD=2.08), venter (M=4.50, SD=2.69), and dorsal pattern (M=7.53, SD=2.78)) pictures/video frames per species, b) assessed observer variability, c) avoided over/under exposed areas, d) assessed the relationship between brightness data obtained from image-analyses and spectrophotometry, e) assessed the variability of image brightness within species, and f) verified the relationship between the visible (Vis), near infrared (NIR) and Ultraviolet (UV) spectra.
Randomization	Our species selection covers ~35% of the family Viperidae and species are equally distributed across the phylogenetic tree.
Blinding	We found positive relationship between the observers (Obs1 vs Obs2: $r = 0.94$; $R^2 = 0.88$; $p < 2.2e-16$, Obs1 vs Obs3: $r = 0.99$; $R^2 = 0.98$; $p = 1.1e-10$, Obs1 vs Obs4: $r = 0.99$; $R^2 = 0.98$; $p = 3.4e-09$, Figure S.3), so only one proceeded with data collection.

Did the study involve field work? ☐ Yes ☒ No

Reporting for specific materials, systems and methods

We require information from authors about some types of materials, experimental systems and methods used in many studies. Here, indicate whether each material, system or method listed is relevant to your study. If you are not sure if a list item applies to your research, read the appropriate section before selecting a response.

Materials & experimental systems

n/a	Involved in the study
☒	☐ Antibodies
☒	☐ Eukaryotic cell lines
☒	☐ Palaeontology and archaeology
☐	☒ Animals and other organisms
☒	☐ Human research participants
☒	☐ Clinical data
☒	☐ Dual use research of concern

Methods

n/a	Involved in the study
☒	☐ ChIP-seq
☒	☐ Flow cytometry
☒	☐ MRI-based neuroimaging

Animals and other organisms

Policy information about studies involving animals; ARRIVE guidelines recommended for reporting animal research

Laboratory animals	Only for spectrophotometry analysis we measured 29 living squamate species, listed in Supplementary File, from the Tel Aviv University's Garden for Zoological Research.
Wild animals	N.A.
Field-collected samples	All animals belong to the collection of the Tel Aviv University's Garden for Zoological Research.
Ethics oversight	Spectrophotometry does not require any invasive procedure. As such, the local staff granted us permission to acquire reflectance measurements under the supervision of the reptile keeper.

Note that full information on the approval of the study protocol must also be provided in the manuscript.
